# Synthesis of novel 6,7-dimethoxy-4-anilinoquinolines as potent c-Met inhibitors

**DOI:** 10.1080/14756366.2018.1533822

**Published:** 2018-11-13

**Authors:** Qing-Wen Zhang, Zi-Dan Ye, Chang Shen, Hong-Xia Tie, Lei Wang, Lei Shi

**Affiliations:** Jiangsu Key Laboratory of Drug Design and Optimization, Department of Medicinal Chemistry, China Pharmaceutical University, Nanjing, P. R. China

**Keywords:** c-Met, inhibitor, 6,7-Dimethoxy-4-anilinoquinoline, synthesis, antitumour

## Abstract

HGF/c-Met signalling pathway plays an important role in the development of cancers. A series of 6,7-dimethoxy-4-anilinoquinolines possessing benzimidazole moiety were synthesised and identified as potent inhibitors of the tyrosine kinase c-Met. Their *in vitro* biological activities against three cancer cell lines (A549, MCF-7, and MKN-45) were also evaluated. Most of these compounds exhibited moderate to remarkable potency. Among them, compound **12n** showed the most potent inhibitory activity against c-Met with IC_50_ value of 0.030 ± 0.008 µM and it also showed excellent anticancer activity against the tested cancer cell lines at low micromolar concentration. Molecular docking verified the results and revealed the possible binding mode of the most promising compound **12n** into the ATP-binding site of c-Met kinase.

## 1. Introduction

The cellular mesenchymal to epithelial transition factor (c-Met), also termed as Met or hepatocyte growth factor receptor (HGFR), is a member of receptor tyrosine kinase which encoded by c-Met proto-oncogene^1–3^. Hepatocyte growth factor (HGF), also known as scatter factor (SF), is the only known high-affinity natural ligand of c-Met. Binding of HGF to c-Met results in receptor homodimerization and phosphorylation and then c-Met is able to recruit several adaptor proteins that in turn activate a number of pathways, including RAS-RAF-MEK-ERK axis, PI3K-AKT-mTOR cascade, SRC, and STAT3^4–6^.

Under normal physiological condition, HGF/c-Met signalling pathway is involved in cell proliferation, survival, migration, scattering, motility and invasion, and plays important roles in mammalian embryonic development, tissue homeostasis, and wound healing^7–10^. However, when deregulated, the c-Met/HGF pathway leads to tumorigenesis and metastasis^7^. Abnormal activation of c-Met signalling due to gene amplification, rearrangement or mutation, transcriptional regulation as well as autocrine or paracrine ligand stimulation, has been involved in various types of human cancers, such as head, neck, thyroid, lung, gastric, oesophageal, breast, ovarian, pancreatic, prostatic, and colorectal carcinomas^11–19^. Additionally, aberrant c-Met activation has been demonstrated to be associated with the acquired resistance of tumour cells during approved therapies^13^^,^^20^^,^^21^. As a result, c-Met has emerged as a promising target for cancer treatment.

**Figure 1.**
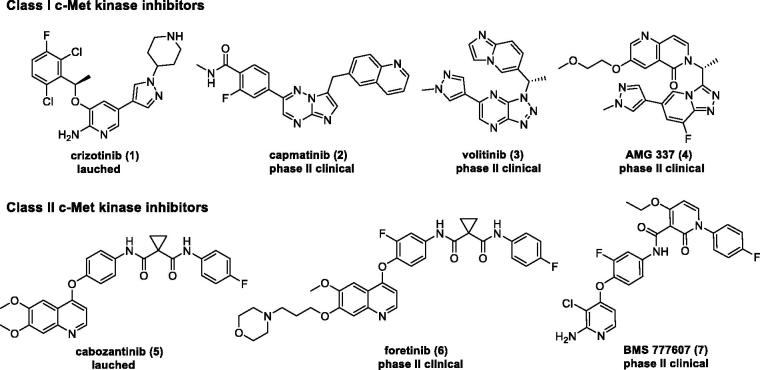
Structures of the representative small-molecule c-Met kinase inhibitors.

**Figure 2.**
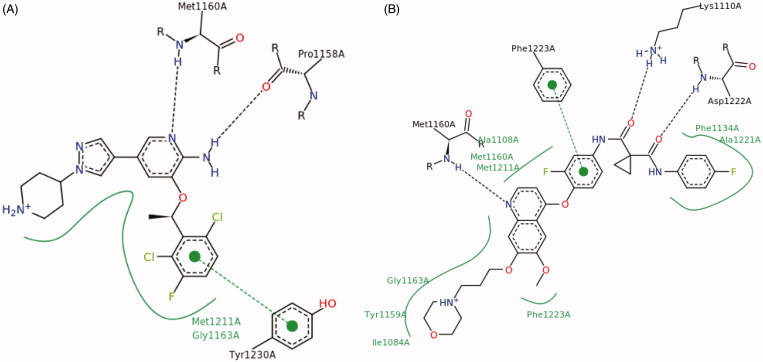
(A) Binding mode of class I inhibitor crizotinib with c-Met. (B) Binding mode of class II inhibitor foretinib with c-Met.

In the past few years, different strategies have been pursued to inhibit abnormal c-Met signalling pathway, including HGF and c-Met biological antagonists^22^^,^^23^, antibodies against HGF or c-Met^24^^,^^25^, and small-molecule c-Met inhibitors^26–28^. Among them, ATP-competitive small-molecule c-Met kinase inhibitor has obtained remarkable achievements within the pharmaceutical industry, leading to the marketing of crizotinib (**1**) and cabozantinib (**5**), and dozens of candidates currently under clinical trials, such as capmatinib (**2**), volitinib (**3**), AMG 337 (**4**), foretinib (**6**), and BMS 777607 (**7**) (Figure 1)^8^. All these small-molecule c-Met inhibitors can basically be categorised into two classes (classes I and II) according to their structures and binding modes with c-Met. Class I c-Met inhibitors (**1**–**4**) bind to the entrance of ATP-binding site in a U-shaped conformation around Met1211, while class II inhibitors (**5**–**7**) bind to c-Met in an extended conformation that stretches from the ATP-binding site to the deep hydrophobic pocket (Figure 2).

**Figure 3.**
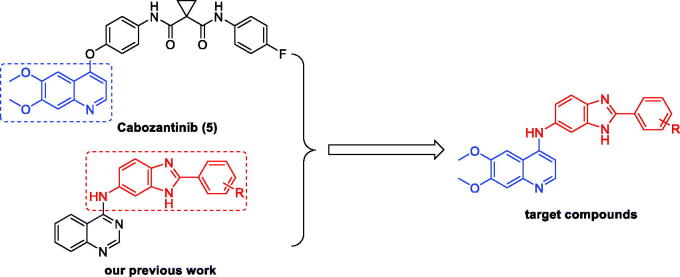
Design strategy for the target compounds.

In our previous studies, a series of N-(2-phenyl-1H-benzo[d]imidazol-5-yl)quinazolin-4-amine derivatives were discovered as potent c-Met inhibitors^29^. In an ongoing effort to pursue novel potent c-Met inhibitors, we modified the previous structures by replacing of the quinazolin fragment with the 6,7-dimethoxyquinolone moiety from cabozantinib (**5**) (Figure 3). All the target compounds were synthesised and evaluated for their inhibitory activities against c-Met kinase and antiproliferative activities against three human cancer cell lines including A549 (human lung cancer), MCF-7 (human breast cancer), and MKN-45 (human gastric cancer). In addition, the structure-activity relationships and docking studies are also presented in this paper.

## 2. Experimental

### 2.1. Chemistry

All reagents and solvents were purchased from commercial sources and used as received without further purification. Reactions were monitored by thin-layer chromatography (TLC) in silica gel and the TLC plates were visualised by exposure to ultraviolet light (254 and 365 nm). Compounds were purified using flash column chromatography over silica gel (200–300 mesh). Melting points (uncorrected) were determined on a RY-1 MP apparatus. ^1^H NMR spectra were recorded on a Bruker AV-300 spectrometer at room temperature with tetramethylsilane (TMS) as an internal standard. Chemical shifts were reported in ppm (*δ*). ESI-MS spectra were recorded on an Agilent/HP 1100 Series LC/MSD Trap SL mass spectrometer. High resolution mass spectrometry (HRMS) was obtained on a Q-tof high resolution mass spectrometer.

#### 2.1.1. General procedure for the preparation of the 5-nitro-2-phenyl-1H-benzo[d]imidazole derivatives (10a-s)

As described in our previous study^29^, a mixture of 4-nitro-*o*-phenylenediamine (21 mmol) and substituted benzoic acid **9a**–**s** (20 mmol) in PPA (40 ml) was stirred at 120–150 °C for 5 h. The reaction was quenched with water and the pH was adjusted to 6 with saturated NaOH. The filter cake was washed with water and recrystallized from ethyl acetate to give corresponding compounds **10a**–**s**.

#### 2.1.2. General procedure for the preparation of the 2-phenyl-1H-benzo[d]imidazol-5-amine derivatives (11a-s)

As described in our previous study^29^, two methods were applied to prepare the intermediates **11a**–**s**.

Method A: A suspension of **10** (5.0 mmol, R=CH_3_, OCH_3_, CH_2_CH_3_, C(CH)_3_) and 10% Pd/C (0.13 g) in methanol (30 mL) was hydrogenated under normal pressure for 5 h at room temperature. Filtration and evaporation gave corresponding compound **11**.

Method B: Compound **10** (5.0 mmol, R=F, Cl, Br, I) and iron (1.1 g, 20 mmol) were suspended in aqueous ethanol (120 mL, 70% v/v) containing acetic acid (2 mL, 30 mmol) and heated at reflux for 2 h. The reaction mixture was cooled to room temperature. Filtration and evaporation gave corresponding compound **11**.

#### 2.1.3. General procedure for the preparation of the 6,7-dimethoxy-N-(2-phenyl-1H-benzo[d]imidazol-6-yl)quinolin-4-amine derivatives (12a-s)

A mixture of 4-chloro-6,7-dimethoxyquinoline (5 mmol) and appropriate amount of substituted anilines (**11a**–**s**, 6 mmol) in isopropanol (40 mL) was stirred at reflux for 5 h. The reaction mixture was concentrated under reduced pressure and the solid residue was purified by column chromatography on silica gel, eluting with CH_2_Cl_2_/CH_3_OH (10/1) to furnish the target compounds **12a**–**s**.

##### 2.1.3.1. N-(2–(2-fluorophenyl)-1H-benzo[d]imidazol-6-yl)-6,7-dimethoxyquinolin-4-amine (12a)

Yellow powder, yield: 54%, mp: 247–249 °C. ^1^H-NMR (300 MHz; DMSO- d_6_): *δ* (ppm) 10.65 (s, 1H), 8.33–8.23 (m, 2H), 8.09 (s, 1H), 7.83 (d, *J* = 8.5 Hz, 1H), 7.73 (d, *J* = 1.8 Hz, 1H), 7.63–7.58 (m, 1H), 7.52–7.43 (m, 2H), 7.40 (d, *J* = 5.3 Hz, 2H), 7.32 (dd, *J* = 8.6, 2.0 Hz, 1H), 6.69 (d, *J* = 7.0 Hz, 1H), 4.01 (s, 3H), 3.99 (s, 3H). HRMS (ESI^+^): calcd C_24_H_20_FN_4_O_2_ (M + H^+^) = 415.1570, found  =  415.1572.

##### 2.1.3.2. N-(2–(3-fluorophenyl)-1H-benzo[d]imidazol-6-yl)-6,7-dimethoxyquinolin-4-amine (12b)

Yellow powder, yield: 55%, mp: 286–288 °C. ^1^H-NMR (300 MHz; DMSO- d_6_): *δ* (ppm) 11.54 (s, 1H), 8.85 (s, 1H), 8.33 (s, 1H), 8.13–8.07 (m, 4H), 7.81 (d, *J* = 9.1 Hz, 1H), 7.69 (s, 2H), 7.48 (d, *J* = 6.8 Hz, 1H), 7.34 (d, *J* = 4.3 Hz, 1H), 4.03 (s, 3H), 4.01 (s, 3H). HRMS (ESI^+^): calcd C_24_H_20_FN_4_O_2_ (M + H^+^) = 415.1570, found  =  415.1576.

##### 2.1.3.3. N-(2–(4-fluorophenyl)-1H-benzo[d]imidazol-6-yl)-6,7-dimethoxyquinolin-4-amine (12c)

Yellow powder, yield: 60%, mp: 312–315 °C. ^1^H-NMR (300 MHz; DMSO- d_6_): *δ* (ppm) 10.56 (s, 1H), 8.15–8.19 (m, 3H), 8.01 (s, 1H), 7.69 (d, *J*  =  8.5 Hz, 1H), 7.59 (s, 1H), 7.38–7.28 (m, 3H), 7.19 (dd, *J* = 8.4, 2.0 Hz, 1H), 6.58 (d, *J* = 7.0 Hz, 1H), 3.91 (s, 3H), 3.89 (s, 3H). HRMS (ESI^+^): calcd C_24_H_20_FN_4_O_2_ (M + H^+^) = 415.1570, found  =  415.1572.

##### 2.1.3.4. N-(2–(2-chlorophenyl)-1H-benzo[d]imidazol-6-yl)-6,7-dimethoxyquinolin-4-amine (12d)

Yellow powder, yield: 51%, mp: 287–290 °C. ^1^H-NMR (300 MHz; DMSO-d_6_): *δ* (ppm) 10.77 (s, 1H), 8.33 (d, *J* = 6.6 Hz, 1H), 8.15 (s, 1H), 7.97 (dd, *J* = 6.9, 2.2 Hz, 1H), 7.88 (d, *J* = 8.6 Hz, 1H), 7.79 (d, *J* = 1.7 Hz, 1H), 7.73 (d, *J* = 7.4 Hz, 1H), 7.61 (td, *J* = 7.1, 1.8 Hz, 2H), 7.42–7.39 (m, 2H), 6.71 (d, *J* = 6.9 Hz, 1H), 4.02 (s, 3H), 4.00 (s, 3H). HRMS (ESI^+^): calcd C_24_H_20_ClN_4_O_2_ (M + H^+^) = 431.1275, found  =  431.1284.

##### 2.1.3.5. N-(2–(3-chlorophenyl)-1H-benzo[d]imidazol-6-yl)-6,7-dimethoxyquinolin-4-amine (12e)

Yellow powder, yield: 46%, mp: 250–252 °C. ^1^H-NMR (300 MHz; DMSO- *d*_6_): *δ* (ppm) 10.67 (s, 1H), 8.35–8.28 (m, 2H), 8.19 (d, *J* = 6.7 Hz, 1H), 8.10 (s, 1H), 7.82 (d, *J* = 8.6 Hz, 1H), 7.73 (s, 1H), 7.63 (d, *J* = 5.3 Hz, 2H), 7.38 (s, 1H), 7.33 (d, *J* = 8.4 Hz, 1H), 6.69 (d, *J* = 7.0 Hz, 1H), 4.01 (s, 3H), 4.00 (s, 3H). HRMS (ESI^+^): calcd C_24_H_20_ClN_4_O_2_ (M + H^+^) = 431.1275, found  =  431.1279.

##### 2.1.3.6. N-(2–(4-chlorophenyl)-1H-benzo[d]imidazol-6-yl)-6,7-dimethoxyquinolin-4-amine (12f)

Yellow powder, yield: 58%, mp: 330–332 °C. ^1^H-NMR (300 MHz; DMSO- d_6_): *δ* (ppm) 13.89 (s, 1H), 10.67 (s, 1H), 8.33 (s, 1H), 8.23 (d, *J* = 8.3 Hz, 2H), 8.11 (s, 1H), 7.79 (d, *J* = 8.5 Hz, 1H), 7.68 (d, *J* = 8.9 Hz, 3H), 7.38 (s, 1H), 7.30 (d, *J* = 8.7 Hz, 1H), 6.68 (d, *J* = 7.0 Hz, 1H), 4.01 (s, 3H), 3.99 (s, 3H). HRMS (ESI^+^): calcd C_24_H_20_ClN_4_O_2_ (M + H^+^) = 431.1275, found  =  431.1276.

##### 2.1.3.7. N-(2–(4-bromophenyl)-1H-benzo[d]imidazol-6-yl)-6,7-dimethoxyquinolin-4-amine (12g)

Yellow powder, yield: 64%, mp: 280–282 °C. ^1^H-NMR (300 MHz; DMSO- d_6_): *δ* (ppm) 10.70 (s, 1H), 8.34 (d, *J* = 7.0 Hz, 1H), 8.20–8.14 (m, 3H), 7.82 (d, *J* = 8.2 Hz, 3H), 7.72 (s, 1H), 7.42 (d, *J* = 3.6 Hz, 1H), 7.31 (d, *J* = 8.3 Hz, 1H), 6.70 (d, *J* = 6.9 Hz, 1H), 4.03 (s, 3H), 4.01 (s, 3H). HRMS (ESI^+^): calcd C_24_H_20_BrN_4_O_2_ (M + H^+^) = 475.0770, found  =  475.0773.

##### 2.1.3.8. N-(2–(2-iodophenyl)-1H-benzo[d]imidazol-6-yl)-6,7-dimethoxyquinolin-4-amine (12h)

Brown powder, yield: 53%, mp: 207–209 °C. ^1^H-NMR (300 MHz; DMSO- d_6_): *δ* (ppm) 12.69 (s, 1H), 8.76 (s, 1H), 8.24 (s, 1H), 8.07 (d, *J* = 7.8 Hz, 1H), 7.73 (s, 1H), 7.66 (d, *J* = 6.6 Hz, 2H), 7.57 (t, *J* = 7.5 Hz, 2H), 7.24 (s, 3H), 6.77 (s, 1H), 3.95 (s, 3H), 3.91 (s, 3H). HRMS (ESI^+^): calcd C_24_H_20_IN_4_O_2_ (M + H^+^) = 523.0631, found  =  523.0639.

##### 2.1.3.9. N-(2–(3-iodophenyl)-1H-benzo[d]imidazol-6-yl)-6,7-dimethoxyquinolin-4-amine (12i)

Brown powder, yield: 60%, mp: 201–203 °C. ^1^H-NMR (300 MHz; DMSO- d_6_): *δ* (ppm) 13.76 (s, 1H), 10.60 (s, 1H), 8.58 (s, 1H), 8.33 (s, 1H), 8.23 (d, *J* = 8.0 Hz, 1H), 8.07 (s, 1H), 7.90 (d, *J* = 8.0 Hz, 1H), 7.80 (d, *J* = 8.4 Hz, 1H), 7.70 (s, 1H), 7.40–7.29 (m, 3H), 6.68 (d, *J* = 7.0 Hz, 1H), 4.00 (s, 3H), 4.00 (s, 3H). HRMS (ESI^+^): calcd C_24_H_20_IN_4_O_2_ (M + H^+^) = 523.0631, found  =  523.0636.

##### 2.1.3.10. 6,7-Dimethoxy-N-(2-(o-tolyl)-1H-benzo[d]imidazol-6-yl)quinolin-4-amine (12j)

Yellow powder, yield: 48%, mp: 174–175 °C. ^1^H-NMR (300 MHz; DMSO- d_6_): *δ* (ppm) 10.87 (s, 1H), 8.37 (s, 1H), 8.21 (s, 1H), 7.91 (d, *J* = 8.6 Hz, 1H), 7.83 (d, *J* = 6.9 Hz, 2H), 7.55–7.43 (m, 5H), 6.75 (d, *J* = 6.9 Hz, 1H), 4.04 (s, 3H), 4.01 (s, 3H), 2.65 (s, 3H). HRMS (ESI^+^): calcd C_25_H_23_N_4_O_2_ (M + H^+^) = 411.1821, found  =  411.1828.

##### 2.1.3.11. 6,7-Dimethoxy-N-(2-(m-tolyl)-1H-benzo[d]imidazol-6-yl)quinolin-4-amine (12k)

Yellow powder, yield: 67%, mp: 339–342 °C. ^1^H-NMR (300 MHz; DMSO- d_6_): *δ* (ppm) 13.91 (s, 1H), 10.66 (s, 1H), 8.35 (s, 1H), 8.08 (d, *J* = 4.5 Hz, 2H), 8.02 (d, *J* = 7.7 Hz, 1H), 7.82 (d, *J* = 8.5 Hz, 1H), 7.73 (s, 1H), 7.51 (t, *J* = 7.7 Hz, 1H), 7.42–7.34 (m, 3H), 6.70 (d, *J* = 7.0 Hz, 1H), 4.01 (s, 3H), 4.00 (s, 3H), 2.44 (s, 3H). HRMS (ESI^+^): calcd C_25_H_23_N_4_O_2_ (M + H^+^) = 411.1821, found  =  411.1819.

##### 2.1.3.12. 6,7-Dimethoxy-N-(2-(p-tolyl)-1H-benzo[d]imidazol-6-yl)quinolin-4-amine (12l)

Yellow powder, yield: 75%, mp: 316–319 °C. ^1^H-NMR (300 MHz; DMSO- d_6_): *δ* (ppm) 10.70 (s, 1H), 8.35 (d, *J* = 6.2 Hz, 1H), 8.15–8.11 (m, 3H), 7.83 (d, *J* = 8.6 Hz, 1H), 7.74 (d, *J* = 1.9 Hz, 1H), 7.45 (d, *J* = 8.0 Hz, 2H), 7.39 (s, 1H), 7.36 (d, *J* = 8.8 Hz, 1H), 6.71 (d, *J* = 7.0 Hz, 1H), 4.01 (s, 3H), 3.99 (s, 3H), 2.41 (s, 3H). HRMS (ESI^+^): calcd C_25_H_23_N_4_O_2_ (M + H^+^) = 411.1821, found  =  411.1827.

##### 2.1.3.13. N-(2–(4-ethylphenyl)-1H-benzo[d]imidazol-6-yl)-6,7-dimethoxyquinolin-4-amine (12m)

Yellow powder, yield: 42%, mp: 210–212 °C. ^1^H-NMR (300 MHz; DMSO- d_6_): *δ* (ppm) 10.66 (s, 1H), 8.34 (s, 1H), 8.15 (d, *J* = 8.0 Hz, 2H), 8.10 (s, 1H), 7.82 (d, *J* = 8.5 Hz, 1H), 7.72 (s, 1H), 7.47 (d, *J* = 8.0 Hz, 2H), 7.38–7.36 (m, 2H), 6.70 (d, *J* = 7.0 Hz, 1H), 4.01 (s, 3H), 4.00 (s, 3H), 2.72 (q, *J* = 7.8 Hz, 2H), 1.25 (t, *J* = 7.6 Hz, 3H). HRMS (ESI^+^): calcd C_26_H_25_N_4_O_2_ (M + H^+^) = 425.1978, found  =  425.1980.

##### 2.1.3.14. N-(2–(4-(tert-butyl)phenyl)-1H-benzo[d]imidazol-6-yl)-6,7-dimethoxyquinolin-4-amine (12n)

Yellow powder, yield: 38%, mp: 257–259 °C. ^1^H-NMR (300 MHz; DMSO- d_6_): *δ* (ppm) 10.79 (s, 1H), 8.35 (s, 1H), 8.21 (d, *J* = 8.1 Hz, 2H), 8.16 (s, 1H), 7.86 (d, *J* = 8.6 Hz, 1H), 7.78 (s, 1H), 7.68 (d, *J* = 8.2 Hz, 2H), 7.42 (s, 2H), 6.73 (d, *J* = 7.0 Hz, 1H), 4.02 (s, 3H), 3.99 (s, 3H), 1.35 (s, 9H). HRMS (ESI^+^): calcd C_28_H_29_N_4_O_2_ (M + H^+^) = 453.2291, found  =  453.2294.

##### 2.1.3.15. 6,7-Dimethoxy-N-(2–(2-methoxyphenyl)-1H-benzo[d]imidazol-6-yl)quinolin-4-amine (12o)

Brown powder, yield: 74%, mp: 164–166 °C. ^1^H-NMR (300 MHz; DMSO- d_6_): *δ* (ppm) 8.79–8.75 (m, 1H), 8.35–8.32 (m, 1H), 8.25 (s, 1H), 7.75 (s, 1H), 7.67 (d, *J* = 8.6 Hz, 1H), 7.58 (s, 1H), 7.47 (d, *J* = 8.6 Hz, 1H), 7.26 (d, *J* = 8.9 Hz, 2H), 7.19–7.10 (m, 1H), 6.80 (s, 1H), 4.04 (s, 3H), 3.95 (s, 3H), 3.91 (s, 3H). HRMS (ESI^+^): calcd C_25_H_23_N_4_O_3_ (M + H^+^) = 427.1770, found  =  427.1776.

##### 2.1.3.16. N-(2–(2,6-difluorophenyl)-1H-benzo[d]imidazol-6-yl)-6,7-dimethoxyquinolin-4-amine (12p)

Yellow powder, yield: 55%, mp: 208–210 °C. ^1^H-NMR (300 MHz; DMSO- d_6_): *δ* (ppm) 8.77 (s, 1H), 8.23 (d, *J* = 5.3 Hz, 1H), 7.74–7.62 (m, 3H), 7.54 (s, 1H), 7.34 (t, *J* = 8.4 Hz, 2H), 7.23 (d, *J* = 7.5 Hz, 2H), 6.72 (d, *J* = 5.4 Hz, 1H), 3.95 (s, 3H), 3.91 (s, 3H). HRMS (ESI^+^): calcd C_24_H_19_F_2_N_4_O_2_ (M + H^+^) = 433.1476, found  =  433.1482.

##### 2.1.3.17. N-(2–(2,6-dichlorophenyl)-1H-benzo[d]imidazol-6-yl)-6,7-dimethoxyquinolin-4-amine (12q)

Yellow powder, yield: 57%, mp: 254–257 °C. ^1^H-NMR (300 MHz; DMSO- d_6_): *δ* (ppm) 8.76 (s, 1H), 8.24 (d, *J* = 5.2 Hz, 1H), 7.72–7.52 (m, 6H), 7.24–7.21 (m, 2H), 6.73 (d, *J* = 5.3 Hz, 1H), 3.95 (s, 3H), 3.91 (s, 3H). HRMS (ESI^+^): calcd C_24_H_19_Cl_2_N_4_O_2_ (M + H^+^) = 465.0885, found  =  465.0890.

##### 2.1.3.18. N-(2–(3,4-dichlorophenyl)-1H-benzo[d]imidazol-6-yl)-6,7-dimethoxyquinolin-4-amine (12r)

Yellow powder, yield: 52%, mp: 336–338 °C. ^1^H-NMR (300 MHz; DMSO- d_6_): *δ* (ppm) 10.73 (s, 1H), 8.48 (d, *J* = 2.0 Hz, 1H), 8.33 (d, *J* = 6.5 Hz, 1H), 8.22 (dd, *J* = 8.4, 2.0 Hz, 1H), 8.13 (s, 1H), 7.89 (d, *J* = 8.4 Hz, 1H), 7.83 (d, *J* = 8.5 Hz, 1H), 7.75–7.69 (m, 1H), 7.42 (d, *J* = 3.9 Hz, 1H), 7.35 (dd, *J* = 8.5, 1.9 Hz, 1H), 6.69 (d, *J* = 7.0 Hz, 1H), 4.01 (s, 3H), 3.99 (s, 3H). HRMS (ESI^+^): calcd C_24_H_19_Cl_2_N_4_O_2_ (M + H^+^) = 465.0885, found  =  465.0884.

##### 2.1.3.19. N-(2–(2-bromo-5-fluorophenyl)-1H-benzo[d]imidazol-6-yl)-6,7-dimethoxyquinolin-4-amine (12s)

Brown powder, yield: 48%, mp: 284–286 °C. ^1^H-NMR (300 MHz; DMSO- d_6_): *δ* (ppm) 10.79 (s, 1H), 8.33 (t, *J* = 6.6 Hz, 1H), 8.18 (s, 1H), 7.94–7.85 (m, 2H), 7.78–7.73 (m, 2H), 7.48–7.37 (m, 3H), 6.70 (d, *J* = 7.0 Hz, 1H), 4.02 (s, 3H), 3.99 (s, 3H). HRMS (ESI^+^): calcd C_24_H_19_BrFN_4_O_2_ (M + H^+^) = 493.0675, found  =  493.0685.

### 2.2. c-Met Inhibition assay

A Caliper motility shift assay was applied to test the potency of synthesised compounds against c-Met kinase. Briefly, the compounds were dissolved in DMSO (Sigma) at 0.5 mM concentration, and then were diluted 50× to the final desired highest inhibitor concentration (10 μM) in reaction by DMSO. For all compounds, 100 μL of the diluted compound solution in tubes was transferred to a well on a 96-well plate (Corning) and was serially diluted 10× by transferring 10 μL dilution to 90 μL DMSO in the next well. 100 μL DMSO was added to two empty wells in the same 96-well plate for no compound control and no enzyme control. The plate was marked as source plate. 10 μL of compound solution from source plate was transferred to a new 96-well plate as the intermediate plate. Then, 90 μL of 1× kinase base buffer (50 mM HEPES, pH 7.5, 0.0015% Brij-35, 10 mM MgCl_2_, 2 mM DTT) was added to each well of the intermediate plate and mixed for 10 min on a shaker. After that, 5 μL solution from each well of the intermediate plate was transferred to a 384-well plate (Corning) as the assay plate in duplicates.c-Met kinase (Carna) was added in 1× kinase base buffer to prepare 2.5× enzyme solution, and FAM-labled peptide (GL Biochem) and ATP (Sigma) were added in 1× kinase base buffer to prepare 2.5× peptide solution. 10 μL of 2.5× enzyme solution was added to each well of the 384-well assay plate that already contained 5 μL of compound in 10% DMSO, and then incubated at room temperature for 10 min. Subsequently, 10 μL of 2.5× peptide solution was added to each well and incubated at 28 °C for specified period of time. After that, 25 μL of stop buffer (100 mM HEPES, pH 7.5, 0.015% Brij-35, 0.2% Coating Reagent, 50 mM EDTA) was added to stop reaction. The data were collected on Caliper and the conversion data were copied from Caliper program.

Inhibition values were obtained according to the following formula: percent inhibition = (max-conversion)/(max-min) × 100, in which “max” stands for DMSO control while “min” stands for no enzyme activity control. IC_50_ values were calculated from three independent experiments by fitting the data in XLfit with the following equation: Y = Bottom + (Top-Bottom)/(1 + (IC_50_/X)^HillSlope).

### 2.3. Cell proliferation assay

The anti-proliferative activities of the prepared compounds (**12a**-**12s**) were evaluated against A549, MCF-7 and MKN-45 cell lines by MTT assay *in vitro*. Briefly, tumour cells were cultured to log phase in RPMI 1640 medium supplemented with 10% foetal bovine serum (FBS). After diluting to 2 × 10^4^ cells mL^−1^ with the complete medium, 100 µL of the obtained cell suspension was added to each well of 96-well culture plates. Subsequently, incubation was performed at 37 °C in 5% CO_2_ for 24 h before the anti-proliferative assessment. Tested samples at pre-set concentrations were added to 6 wells with cabozantinib as a positive reference. After 48 h exposure period, 40 µL of PBS containing 2.5 mg mL^−1^ of MTT was added to each well. Four hours later, 100 µL extraction solution (10% SDS-5% isobutyl alcohol-0.010M HCl) was added. After an overnight incubation at 37°C, the absorbance was measured at a wavelength of 570 nm with an ELISA microplate reader. All compounds were tested three times in each tumour cell lines. The IC_50_ values were the averages of three determinations and calculated by concentration-response curve fitting method.

### 2.4. Molecular docking

Molecular docking of compounds **12a**–**s** into the three dimensional X-ray structure of c-Met kinase (PDB code: 3CD8)^30^ was carried out using the Discovery Stutio (version 4.0)^31^ as implemented through the graphical user interface Discovery Studio CDOCKER protocol. The three-dimensional structures of the compounds **12a**–**s** were constructed using ChemBio 3 D Ultra 14.0 software [Chemical Structure Drawing Standard; Cambridge Soft corporation, USA (2014)], then they were energetically minimised by using MMFF94. The crystal structures of c-Met kinase were retrieved from the RCSB Protein Data Bank (http://www.rcsb.org). All bound waters and ligands were eliminated from the protein and the polar hydrogen was added. The whole 3CD8 was defined as a receptor and the site sphere was selected based on ATP binding site of 3CD8. Compounds **12a**–**s** were placed during the molecular docking procedure. Types of interactions of the docked protein with ligand were analysed after the end of molecular docking.

## 3. Results and discussion

### 3.1. Chemistry

**Scheme 1.**
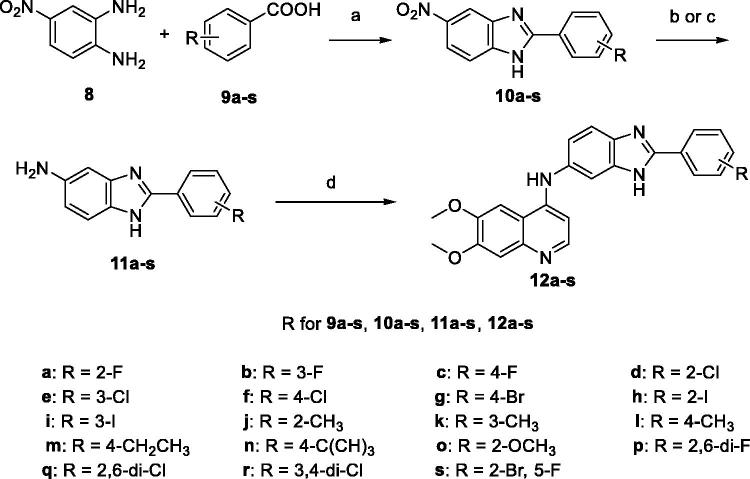
General procedure for the synthesis of 6,7-dimethoxy-4-anilinoquinolines. Reagents and conditions: (a) PPA, 120–150 °C, 5 h; (b) Pd/C, H_2_, CH_3_OH, r.t., 5 h; (c) Fe, AcOH, EtOH, reflux, 2 h; (d) 4-chloro-6,7-dimethoxyquinoline, isopropanol, reflux, 5 h.

The route employed to synthesise the title 6,7-dimethoxy-4-anilinoquinolones bearing benzimidazole moiety is outlined in Scheme 1, which was illustrated in detail in our previous study^29^. Briefly, the preparation of the target compounds started from a series of commercially available substituted benzoic acids **9a**–**s**. Condensation of **9a**–**s** with 4-nitrobenzene-1,2-diamine (**8**) in polyphosphoric acid (PPA) at 120–150 °C for 5 h gave the 5-nitro-2-substituted-phenyl-benzimidazole intermediates **10a**–**s**. Hydrogenation of 5-nitro-2-substituted-phenyl-benzimidazole intermediates **10**–**s** with Pd/C/H_2_ in methanol under normal pressure at room temperature for 5 h or Fe/AcOH in ethanol under reflux for 2 h provided the 5-amino–2-substituted-phenyl-benzimidazole intermediates **11a**–**s**. Condensation of **11a**–**s** with 4-chloro-6,7-dimethoxyquinoline in isopropanol under reflux for 5 h gave the target compounds **12a–s**.

### 3.2. Biological evaluation

#### 3.2.1. *In vitro* c-Met kinase assay

All the synthesised 6,7-dimethoxy-*N*-(2-phenyl-1*H*-benzo[*d*]imidazol-6-yl)quinolin-4-amine derivatives **12a**–**s** were evaluated for their inhibitory activity against c-Met kinase using Caliper motility shift assay. The results were summarised in Table 1 with cabozantinib as a positive control.

**Table 1.** Chemical structures of target compounds and their c-Met inhibitory activities *in vitro*. 
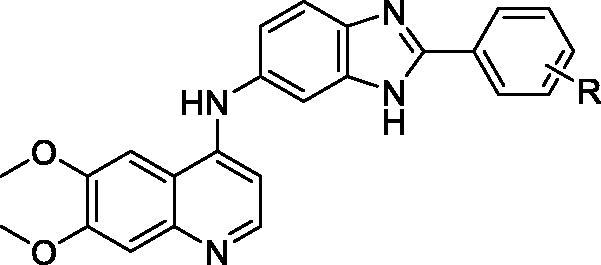

Compound	R	c-Met inhibition	
		% Inhibition (10 μΜ)^a^	IC_50_ (μM)^a^
**12a**	2-F	16.9 ± 3.5	N.D.^b^
**12b**	3-F	62.8 ± 1.2	1.2 ± 0.4
**12c**	4-F	89.2 ± 0.3	0.11 ± 0.02
**12d**	2-Cl	17.4 ± 3.7	N.D.
**12e**	3-Cl	54.5 ± 6.9	4.8 ± 0.5
**12f**	4-Cl	63.8 ± 0.7	0.93 ± 0.10
**12g**	4-Br	54.2 ± 0.1	5.5 ± 0.9
**12h**	2-I	21.6 ± 2.5	N.D.
**12i**	3-I	66.0 ± 0.8	0.54 ± 0.13
**12j**	2-CH_3_	23.0 ± 0.4	N.D.
**12k**	3-CH_3_	52.7 ± 1.6	8.5 ± 2.0
**12l**	4-CH_3_	69.0 ± 1.2	0.32 ± 0.07
**12m**	4-CH_2_CH_3_	95.0 ± 0.3	0.056 ± 0.012
**12n**	4-C(CH_3_)_3_	97.1 ± 3.5	0.030 ± 0.008
**12o**	2-OCH_3_	24.2 ± 0.1	N.D.
**12p**	2,6-di-F	25.9 ± 1.5	N.D.
**12q**	2,6-di-Cl	37.0 ± 4.0	N.D.
**12r**	3,4-di-Cl	55.4 ± 2.9	5.3 ± 1.6
**12s**	2-Br-5-F	23.0 ± 1.1	N.D.
**cabozantinib**^c^		N.D.	0.0045 ± 0.0006

a *n* = 3 (mean ± SD).

b N.D.: not determined.

c Used as a positive control.

As illustrated in Table 1, some of these novel 6,7-dimethoxy-4-anilinoquinolones bearing benzimidazole fragment were found to be active against c-Met kinase. Among them, compound **12n** exhibited the most potent inhibitory activity against c-Met with IC_50_ value of 0.030 ± 0.008 μM.

Structure–activity relationships (SARs) were inferred from the data shown in Table 1. Various functional groups were introduced to the phenyl ring attached to the benzimidazole fragment, which played important roles in the optimisation of c-Met inhibitory potency.

Compounds **12a–c** with fluoro substituent at different position of the phenyl ring showed distinct inhibitory activity against c-Met. Compound **12c** with *para*-fluoro substituent showed 11-fold more potent activity (IC_50_ = 0.11 ± 0.02 μM) than compound **12 b** with *meta*-fluoro substituent (IC_50_ = 1.2 ± 0.4 μM), whereas *ortho*- fluoro substitution (**12a**) led to diminished activity. The result suggested that substituent at different positions led to different inhibitory activities in the following order: *para-* > *meta*- > *ortho*-. This rule was also found in other single substituted compounds (**12d***vs***12e** and **12f**, **12 h***vs***12i**, **12j***vs***12k** and **12l**).

The inhibitory activities against c-Met of compounds with different *para*-substituents on the phenyl ring declined in the following order: compound **12n** with *para*- *tert*-butyl substituent exhibited the most potent inhibitory activity against c-Met (IC_50_ = 0.030 ± 0.008 μM); compound **12 m** with *para*-ethyl substituent displayed slightly weaker inhibitory activity (IC_50_ = 0.056 ± 0.012 μM); compound **12 l** with *para*-methyl substituent showed much weaker inhibitory activity (IC_50_ = 0.32 ± 0.07 μM). Besides, compounds **12c**, **12f** and **12 g** with different *para*-halogen substituents on the phenyl ring presented distinguishing inhibitory activities against c-Met: compound **12c** with *para-*fluoro substituent displayed potent activity (IC_50_ = 0.11 ± 0.02 μM); compound **12f** with *para-*chloro substituent exhibited moderate activity (IC_50_ = 0.93 ± 0.10 μM), which was about 9-fold lower than compound **12c**; compound **12 g** with *para-*bromo substituent showed mild activity (IC_50_ = 5.5 ± 0.9 μM), which was about 50-fold lower than compound **12c**. The above results indicated that introduction of bulk lipophilic substituent is favourable for the activity, probably due to that it can form hydrophobic interaction with the active site of c-Met kinase.

Apart from single substituted compounds (**12a**–**o**), we also synthesised several compounds with double halogen substituents on the phenyl ring (**12p**–**s**). Compound **12r** with two chloro substituents at *para* and *meta* positions displayed mild inhibitory activity against c-Met (IC_50_ = 5.3 ± 1.6 μM). However, as long as one *ortho* position of the phenyl ring was occupied by any halogen substituent (e.g. **12p**, **12q** and **12s**), it resulted in the loss of activity. The above results also demonstrated that introduction of substituents at *ortho* position of the phenyl ring was unfavourable for the activity.

#### 3.2.2. *In vitro* antiproliferation assay

All the synthesised compounds **12a**–**n** were evaluated for their antiproliferation activities against A549 (human lung cancer), MCF-7 (human breast cancer) and MKN-45 (human gastric cancer) cell lines by MTT assay, using cabozantinib as the positive control. The results expressed as IC_50_ values were presented in Table 2. Among the tested compounds, compound **12n** with the most potent c-Met inhibitory activity also displayed the most potent anticancer activities against A549, MCF-7 and MKN-45 with IC_50_ values of 7.3 ± 1.0 μM, 6.1 ± 0.6 μM, and 13.4 ± 0.5 μM, respectively, which were comparable to the reference drug cabozantinib. Basically, the SARs analysis result of antiproliferation activities of the tested compounds were consistent with that of their inhibitory activities against c-Met kinase, which suggested that the potent anticancer activities of the synthesised compounds were probably related to their c-Met inhibitory activities.

**Table 2.** Chemical structures of target compounds and their antiproliferation activities against A549, MCF-7, and MKN-45 cell lines *in vitro*. 
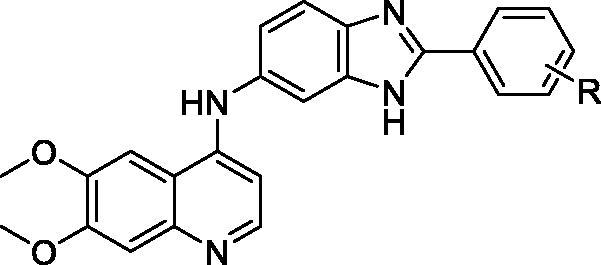

Compound	R	Proliferative inhibition (IC_50_, µM)^a^		
		A549	MCF-7	MKN-45
**12a**	2-F	>100	>100	>100
**12b**	3-F	45.0 ± 3.3	48.6 ± 5.2	57.5 ± 1.9
**12c**	4-F	15.6 ± 2.5	11.3 ± 1.6	21.0 ± 1.8
**12d**	2-Cl	>100	>100	>100
**12e**	3-Cl	53.8 ± 5.0	63.5 ± 4.8	85.7 ± 3.7
**12f**	4-Cl	32.4 ± 3.6	29.2 ± 1.4	39.5 ± 2.5
**12g**	4-Br	38.5 ± 2.4	46.3 ± 3.7	55.0 ± 3.4
**12h**	2-I	92.4 ± 5.1	>100	>100
**12i**	3-I	23.8 ± 2.0	30.5 ± 1.6	36.1 ± 2.7
**12j**	2-CH_3_	>100	>100	>100
**12k**	3-CH_3_	72.6 ± 6.5	78.3 ± 3.5	87.2 ± 6.7
**12l**	4-CH_3_	27.3 ± 1.6	24.0 ± 0.9	31.8 ± 1.4
**12m**	4-CH_2_CH_3_	12.5 ± 1.3	18.4 ± 2.1	14.9 ± 0.7
**12n**	4-C(CH_3_)_3_	7.3 ± 1.0	6.1 ± 0.6	13.4 ± 0.5
**12o**	2-OCH_3_	>100	>100	>100
**12p**	2,6-di-F	>100	>100	>100
**12q**	2,6-di-Cl	>100	>100	>100
**12r**	3,4-di-Cl	83.6 ± 4.0	79.2 ± 1.7	>100
**12s**	2-Br-5-F	>100	92.7 ± 5.3	>100
**cabozantinib**^b^		4.5 ± 0.8	7.2 ± 0.5	11.8 ± 1.4

a *n* = 3 (mean ± SD).

b Used as a positive control.

### 3.3. Molecular docking studies

To further elucidate the interaction between the synthesised compounds and c-Met kinase, molecular docking of compounds **12a**–**s** into the ATP binding site of c-Met kinase (PDB: 3CD8) was performed using the Discovery Studio 4.0/CDOCKER protocol.

**Figure 4.**
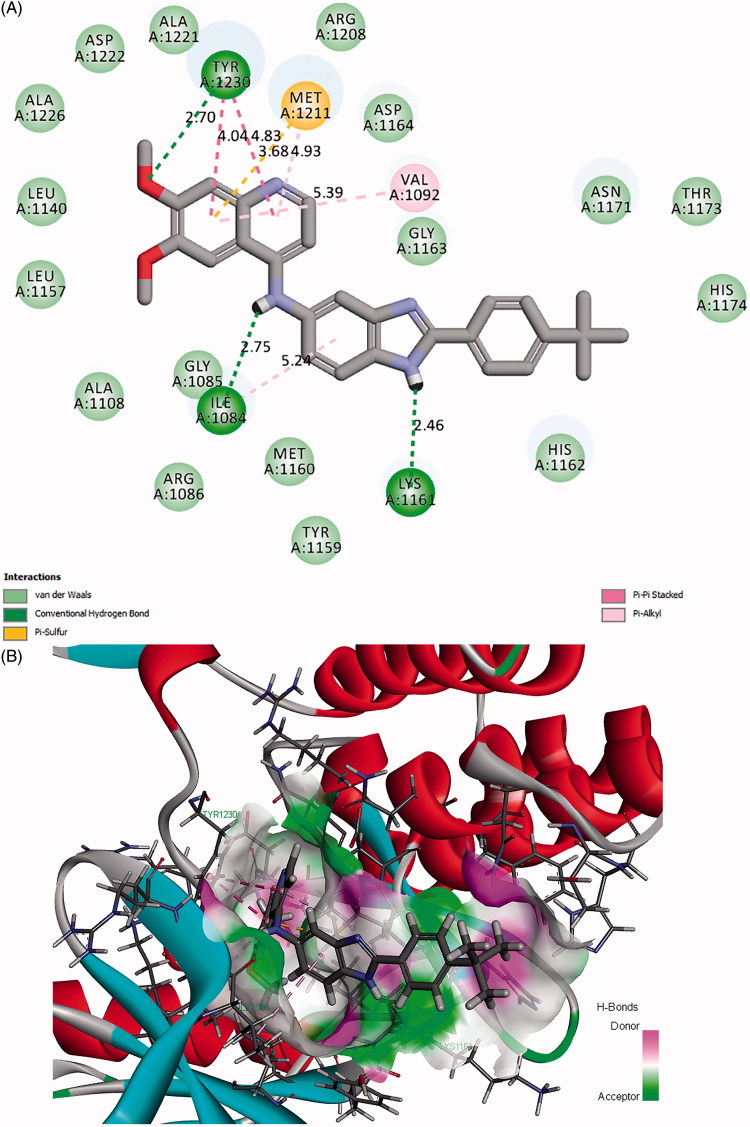
(A) 2D molecular docking modelling of compound **12n** with c-Met kinase. (B) 3D model of the interaction between compound **12n** and c-Met ATP-binding site.

**Figure 5.**
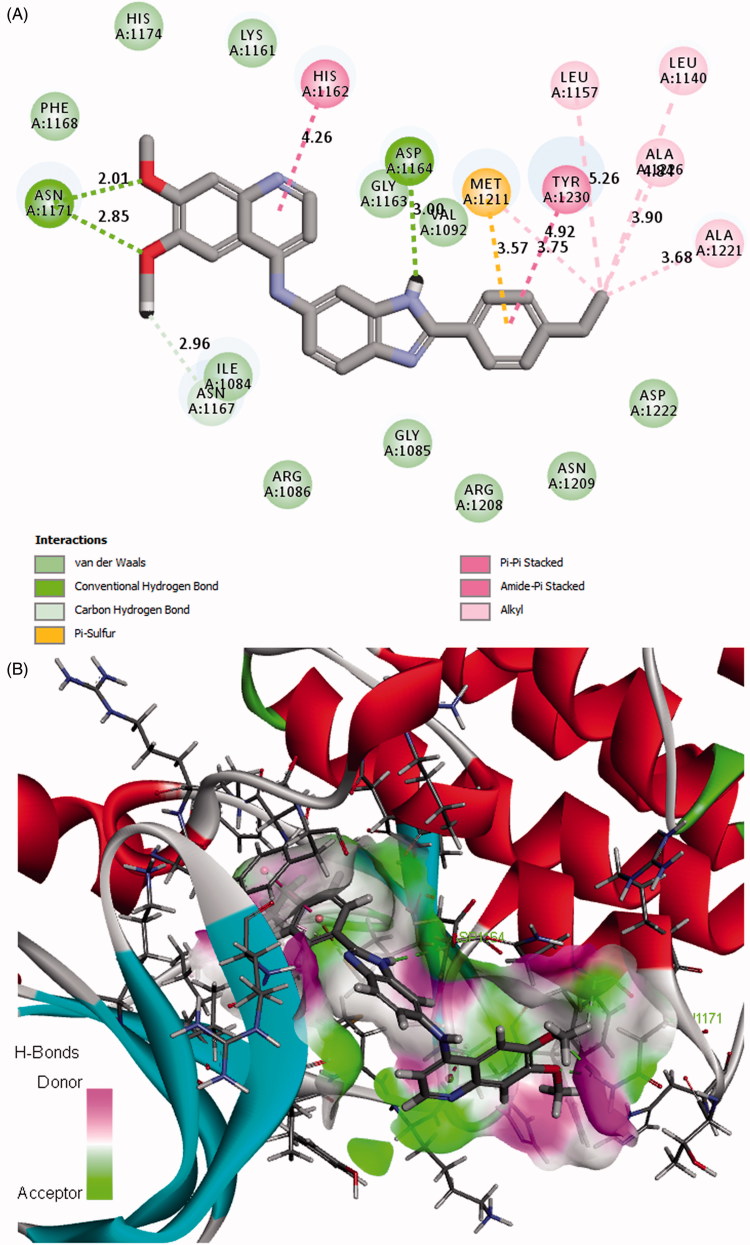
(A) 2D molecular docking modelling of compound **12m** with c-Met kinase. (B) 3D model of the interaction between compound **12m** and c-Met ATP-binding site.

The binding model of the most potent compound **12n** and c-Met is depicted in Figures 4(A) and (B). Visual inspection of the pose of compound **12n** into c-Met ATP-binding site revealed that compound **12n** was tightly embedded into the binding pocket via three conventional hydrogen bonds, two π-π stacked interactions, three π-alkyl interactions one π-sulfur interaction, and many Van der Waals interactions. Specifically, the methoxyl oxygen atom at the quinoline forms a hydrogen bond (O…H-O: 2.70 Å, 117.11°) with the hydroxyl hydrogen atom of TYR1230. The phenyl ring of quinoline forms a π-π stacked interaction (distance: 4.04 Å) with the phenyl ring of TYR1230, a π-alkyl interaction with VAL1092 (distance: 5.39 Å), and a π-sulfur interaction with MET1211 (distance: 3.68 Å) as well. In addition, the pyridine ring of quinoline form another π-π hydrophobic interaction (distance: 4.83 Å) with the phenyl ring of TYR1230 and also forms a π-alkyl interaction with MET1211 (distance: 4.93 Å). These results indicated that the quinoline moiety plays an important role in the combination of the receptor and ligand. Besides, the amino hydrogen atom of benzimidazole forms a hydrogen bond (O…H-N: 2.46 Å, 136.32°) with the carbonyl oxygen atom of LYS1161. The phenyl ring of benzimidazole forms a π-alkyl interaction with ILE1084 (distance: 5.24 Å). These results suggested that the introduction of benzimidazole moiety may be favourable for the interaction between compound **12n** and c-Met, which might be beneficial for the enhancement of the binding affinity, leading to the increased c-Met inhibition and consequent anticancer activity of compound **12n**. Moreover, the hydrogen atom of the amino group which links the qunoline and benzimidazole moieties forms a hydrogen bond with the carbonyl oxygen atom of ILE1084 (O…H-N: 2.75 Å, 116.72^°^). Furthermore, it is noteworthy that the highly lipophilic *tert*-butyl group at the phenyl ring occupies the hydrophobic pocket of c-Met and forms hydrophobic interactions with the amino acid residues nearby, which may contribute to the potency increment of compound **12n** against c-Met. The binding mode of compound **12 m** with comparable activity to **12n** was shown in Figures 5(A) and (B). Compound **12 m** also formed various types of interactions with c-Met, which may contribute to its potency.

## 4. Conclusions

In summary, a series of 6,7-dimethoxy-4-anilinoquinolone derivatives bearing the benzimidazole scaffold have been designed and synthesised as potent inhibitors against c-Met kinase. Their antiproliferation activities against three human cancer cell lines (A549, MCF-7, and MKN-45) have also been evaluated. Compound **12n** showed the most potent inhibitory activity against c-Met with IC_50_ value of 0.030 ± 0.008 μM, and exhibited the strongest inhibitory activities against A549, MCF-7 and MKN-45 cancer cell lines with IC_50_ values of 7.3 ± 1.0 μM, 6.1 ± 0.6 μM, and 13.4 ± 0.5 μM, respectively. Analysis of SARs indicated that introduction of bulk lipophilic substituent to the phenyl ring linking to the benzimidazole moiety is beneficial for the potency of compounds against c-Met. Molecular docking of the most potent inhibitor **12n** into ATP-binding site of c-Met kinase was performed and the result demonstrated that compound **12n** could bind tightly with the active site of c-Met by various interactions. All the above results indicated that compound **12n** could be a potential anticancer agent and deserves further study.

## References

[1] Bottaro DP, Rubin JS, Faletto DL, et al Identification of the hepatocyte growth factor receptor as the c-met proto-oncogene product. Science 1991;251:802–4. 1846706 10.1126/science.1846706

[2] Bertotti A, Comoglio PM Tyrosine kinase signal specificity: lessons from the HGF receptor. Trends Biochem Sci 2003;28:527–33. 14559181 10.1016/j.tibs.2003.09.001

[3] Galimi F, Brizzi MF, Comoglio PM The hepatocyte growth factor and its receptor. Stem Cells Suppl 1996;11:22–30. 10.1002/stem.5530110805 8401259

[4] Ponzetto C, Bardelli A, Zhen Z, et al A multifunctional docking site mediates signaling and transformation by the hepatocyte growth factor/scatter factor receptor family. Cell 1994;77:261–71. 7513258 10.1016/0092-8674(94)90318-2

[5] Furge KA, Zhang YW, Vande Woude GF Met receptor tyrosine kinase: enhanced signaling through adapter proteins. Oncogene 2000;19:5582–9. 11114738 10.1038/sj.onc.1203859

[6] Trusolino L, Comoglio PM Scatter-factor and semaphorin receptors: cell signalling for invasive growth. Nat Rev Cancer 2002;2:289–300. 12001990 10.1038/nrc779

[7] Birchmeier C, Birchmeier W, Gherardi E, et al. Met, metastasis, motility and more. Nat Rev Mol Cell Biol 2003;4:915–25. 14685170 10.1038/nrm1261

[8] Parikh PK, Ghate MD Recent advances in the discovery of small molecule c-Met Kinase inhibitors. Eur J Med Chem 2018;143:1103–38. 29157685 10.1016/j.ejmech.2017.08.044

[9] Trusolino L, Bertotti A, Comoglio PM MET signalling: principles and functions in development, organ regeneration and cancer. Nat Rev Mol Cell Biol 2010;11:834–48. 21102609 10.1038/nrm3012

[10] Chmielowiec J, Borowiak M, Morkel M, et al. c-Met is essential for wound healing in the skin. J Cell Biol 2007;177:151–62. 17403932 10.1083/jcb.200701086 PMC2064119

[11] Lim YC, Kang HJ, Moon JH C-Met pathway promotes self-renewal and tumorigenecity of head and neck squamous cell carcinoma stem-like cell. Oral Oncol 2014;50:633–9. 24835851 10.1016/j.oraloncology.2014.04.004

[12] Ruco L, Scarpino S The pathogenetic role of the HGF/c-Met system in papillary carcinoma of the thyroid. Biomedicines 2014;2:263–74. 28548071 10.3390/biomedicines2040263 PMC5344270

[13] Bean J, Brennan C, Shih JY, et al. MET amplification occurs with or without T790M mutations in EGFR mutant lung tumors with acquired resistance to gefitinib or erlotinib. Proc Natl Acad Sci USA 2007;104:20932–7. 18093943 10.1073/pnas.0710370104 PMC2409244

[14] Houldsworth J, Cordon-Cardo C, Ladanyi M, et al. Gene amplification in gastric and esophageal adenocarcinomas. Cancer Res 1990;50:6417–22. 2400999

[15] Gunasinghe NP, Wells A, Thompson EW, et al. Mesenchymal-epithelial transition (MET) as a mechanism for metastatic colonisation in breast cancer. Cancer Metastasis Rev 2012;31:469–78. 22729277 10.1007/s10555-012-9377-5

[16] Tang C, Jardim DL, Falchook GS, et al. MET nucleotide variations and amplification in advanced ovarian cancer: characteristics and outcomes with c-Met inhibitors. Oncoscience 2013;1:5–13. 25593979 10.18632/oncoscience.3 PMC4295762

[17] Delitto D, Vertes GE, Hughes SJ, et al. c-Met signaling in the development of tumorigenesis and chemoresistance: potential applications in pancreatic cancer. World J Gastroenterol 2014;20:8458–70. 25024602 10.3748/wjg.v20.i26.8458 PMC4093697

[18] Humphrey PA, Zhu X, Zarnegar R, et al. Hepatocyte growth factor and its receptor (c-MET) in prostatic carcinoma. Am J Pathol 1995;147:386–96. 7639332 PMC1869824

[19] Liu Y, Yu XF, Zou J, et al. Prognostic value of c-Met in colorectal cancer: a meta-analysis. World J Gastroenterol 2015;21:3706–10. 25834339 10.3748/wjg.v21.i12.3706 PMC4375596

[20] Engelman JA, Zejnullahu K, Mitsudomi T, et al. Met amplification leads to gefitinib resistance in lung cancer by activating ERBB3 signaling. Science 2007;316:1039–43. 17463250 10.1126/science.1141478

[21] Straussman R, Morikawa T, Shee K, et al. Tumour micro-environment elicits innate resistance to RAF inhibitors through HGF secretion. Nature 2012;487:500–4. 22763439 10.1038/nature11183 PMC3711467

[22] Chan AM, Rubin JS, Bottaro DP, et al. Identification of a competitive HGF antagonist encoded by an alternative transcript. Science 1991;254:1382–5. 1720571 10.1126/science.1720571

[23] Michieli P, Mazzone M, Basilico C, et al. Targeting the tumor and its microenvironment by a dual-function decoy Met receptor. Cancer Cell 2004;6:61–73. 15261142 10.1016/j.ccr.2004.05.032

[24] Burgess T, Coxon A, Meyer S, et al. Fully human monoclonal antibodies to hepatocyte growth factor with therapeutic potentialagainst hepatocyte growth factor/c-Met-dependent human tumors. Cancer Res 2006;66:1721–9. 16452232 10.1158/0008-5472.CAN-05-3329

[25] Martens T, Schmidt NO, Eckerich C, et al. A novel one-armed anti-c-Met antibody inhibits glioblastoma growth in vivo. Clin Cancer Res 2006;12:6144–52. 17062691 10.1158/1078-0432.CCR-05-1418

[26] Ugolini M, Kenigsberg A, Rak F, et al. Discovery, pharmacokinetic and pharmacological properties of the potent and selective MET kinase inhibitor, 1-{6-[6-(4-Fluoro-phenyl)-[1,2,4]triazolo[4,3-b]pyridazin-3-ylsulfanyl]-benzothiazol-2-yl}-3-(2-morpholin-4-yl-ethyl)-urea (SAR125844). J Med Chem 2016;59:7066–74. 27355974 10.1021/acs.jmedchem.6b00280

[27] Tang Q, Wang L, Duan Y, et al. Discovery of novel 7-azaindole derivatives bearing dihydropyridazine moiety as c-Met kinase inhibitors. Eur J Med Chem 2017;133:97–106. 28384549 10.1016/j.ejmech.2017.03.045

[28] Zhang L, Zhao J, Zhang B, et al. Discovery of [1,2,4]triazolo[3,4-b][1,3,4]thiadiazole derivatives as novel, potent and selective c-Met kinase inhibitors: Synthesis, SAR study, and biological activity. Eur J Med Chem 2018;150:809–16. 29602036 10.1016/j.ejmech.2018.03.049

[29] Shi L, Wu TT, Wang Z, et al. Discovery of quinazolin-4-amines bearing benzimidazole fragments as dual inhibitors of c-Met and VEGFR-2. Bioorg Med Chem 2014;22:4735–44. 25082515 10.1016/j.bmc.2014.07.008

[30] Albrecht BK, Harmange JC, Bauer D, et al. Discovery and optimization of triazolopyridazines as potent and selective inhibitors of the c-Met kinase. J Med Chem 2008;51:2879–82. 18426196 10.1021/jm800043g

[31] Accelrys Software Inc. Discovery Studio Visualizer, Release 4.0. Accelrys Software Inc., San Diego, CA, USA, 2013.

